# Ross River Virus: Many Vectors and Unusual Hosts Make for an Unpredictable Pathogen

**DOI:** 10.1371/journal.ppat.1005070

**Published:** 2015-09-03

**Authors:** Suzi B. Claflin, Cameron E. Webb

**Affiliations:** 1 Department of Entomology, Cornell University, Ithaca, New York, United States of America; 2 Department of Medical Entomology, University of Sydney and Pathology West—ICPMR Westmead, Westmead Hospital, Westmead, New South Wales, Australia; University of Kentucky, UNITED STATES

## Introduction

**Fig 1:**
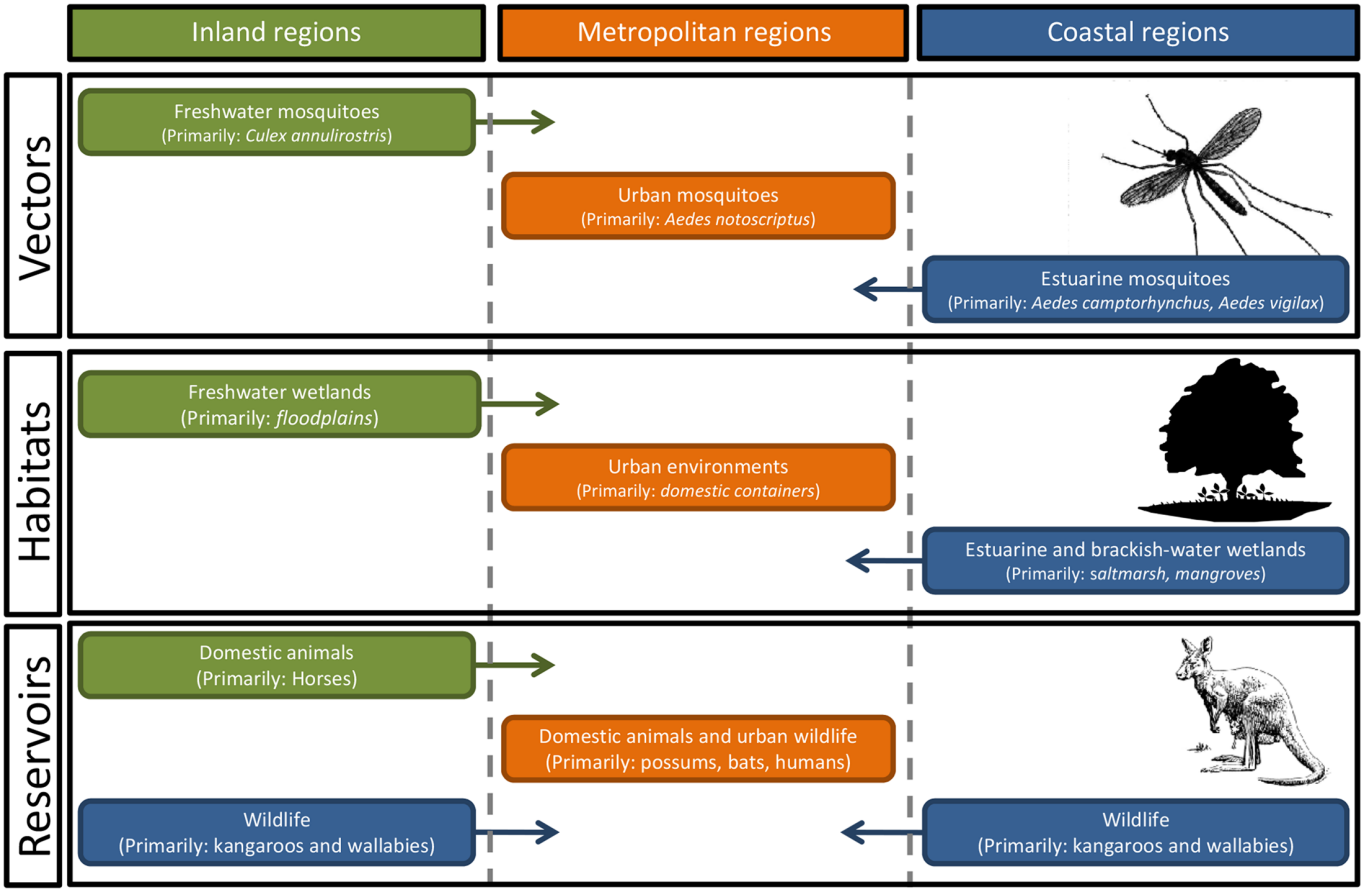
There are complex relationships between the vectors and the zoonotic reservoirs of Ross River virus across coastal, inland, and metropolitan regions of Australia. With around 40 different mosquito species implicated as potential vectors, the environmental drivers of mosquito abundance will vary, not only with the abundance and distribution of habitats within each region but also temporally, with differences in temperature, rainfall, and tidal inundations of estuarine wetlands.

While most mosquito-borne viruses are associated with a narrow range of vector and reservoir host species, some pathogens have much larger vector and host assemblages. One such group is the Alphaviruses (including chikungunya virus [CHIKV]), with Ross River virus (RRV), endemic to Australia, providing a fascinating example of the complicated relationship between vector and reservoir host species across different environments (Fig 1). RRV is responsible for the most commonly reported mosquito-borne disease in Australia, and as both a reservoir host and vector generalist, the virus has complex spatial and temporal activity that makes outbreak prediction, vector and pathogen surveillance, and public health risk mitigation strategies difficult. Here, we review the unique ecology of RRV and the challenges it presents for local health authorities.

## Ross River Virus Disease

Ross River virus disease (also commonly known as Ross River Fever) is not fatal. However, the associated arthralgia can be seriously debilitating. While there are a wide range of disease symptoms, they typically include arthritic joint pain, usually of the peripheral joints, which affects 83%–98% of patients; fatigue and rash, both of which affect over 50% of patients; and fever, which affects 20%–60% of patients [1]. The severity of symptoms varies, as does their duration, which can range from a few weeks to several months [2]; several studies indicate that chronic joint pain affects over 50% of RRV disease patients, which can persist for years after diagnosis [1]. The public health impacts of RRV disease are significant: it is estimated to cost Australia at least US$4.1–US$4.7 million per year [3].

The incidence of RRV disease varies regionally [2]. Roughly 5,000 cases of RRV disease are officially notified each year [4]. There are generally more cases recorded in northern Australia, but the virus still poses a substantial threat in the temperate southern regions of the country. RRV disease case numbers are generally thought to be an underestimate [5]. Accurately quantifying the scope of RRV disease is difficult, as the variability in symptom severity and the requirement of a blood test to confirm infection may cause many milder cases to go undiagnosed. As a consequence, official statistics may only represent the most severe cases.

There are no specific treatments available for the disease; patients are usually given supportive care and prescribed general analgesics and anti-inflammatory agents to treat symptoms [1]. While a vaccine is in development [6], current prevention strategies rely primarily on mosquito avoidance and control.

## Reservoir Hosts

It is rare that a national icon is also a key reservoir host for a mosquito-borne pathogen. Yet, native Australian macropods, such as the beloved kangaroo and wallaby, are currently thought to be the most significant of RRV’s large suite of reservoir hosts, for both the maintenance of the virus in nature and its transmission to humans [7]. Serological studies and laboratory investigations have indicated that several other domestic and wild animals serve as RRV reservoirs, including dogs, cats, possums, and horses [5]. Humans have been implicated as critical reservoir hosts in significant RRV outbreaks across the Pacific Islands [1] and within metropolitan areas in Australia [8], regions where no macropods are present. High viremia in human patients and low seroprevalence in nonhuman vertebrates during outbreaks provide strong evidence for human–mosquito–human transmission in these cases [1]. Under these conditions, RRV is capable of epidemic local spread, as in the South Pacific outbreak of 1979–1989, allowing it to expand its range and re-emerge outside of Australia—regardless of the presence of preferred enzootic hosts—so long as competent mosquito vectors are present.

## Vectors

Ross River virus is a vector generalist. It was first isolated from mosquitoes (*Aedes vigilax*) collected in Queensland in 1959 [9], and since that time, over 40 species of mosquitoes across Australia have been identified that may play a role in transmission. While most mosquito species have been incriminated by the isolation of RRV from field-collected specimens, laboratory vector competence experiments have confirmed effective transmission of the virus by more than ten species [5].

Field surveillance and laboratory testing have identified a diverse range of particularly influential species, including *Aedes camptorhynchus*, *Aedes notoscriptus*, *Aedes vigilax*, and *Culex annulirostris*. *A*. *camptorhynchus* and *A*. *vigilax* are closely associated with estuarine wetlands, with local population abundance determined by local tidal flooding and rainfall inundating habitats. The abundance of *C*. *annulirostris*, associated with freshwater ephemeral and permanent habitats, is determined by rainfall and riverine flooding. Finally, the metropolitan species *A*. *notoscriptus* is associated with water-holding containers [10]. There is also a suite of mosquito species found in freshwater and brackish environments that may play an important role in local enzootic and epidemic transmission. The diverse range of vector habitats, together with concomitant environmental drivers of mosquito abundance across coastal, freshwater, and urban environments, clearly illustrates the ecological complexity of RRV transmission cycles and the challenges faced by those attempting to manage the associated public health risks.

## Management and Surveillance of Public Health Risks

Traditionally, RRV was considered a greater risk in rural regions, where there are both large mosquito populations and an abundance of suitable reservoir hosts. However, recently there have been RRV disease outbreaks on the outskirts of several major metropolitan regions, including Brisbane, Sydney, and Perth [5]. As urbanization of coastal regions increases, human populations will continue to encroach on mosquito and wildlife habitats, increasing the risk of mosquito-borne disease. Mosquito control can be an effective strategy for disease mitigation [11], but few local authorities have the capacity to maintain broadscale treatment programs. In most regions, disease prevention continues to rely heavily on personal protection measures, such as insect repellents, promoted by local health authorities [12].

The diversity of RRV vector species and the associated environmental drivers of vector abundance create spatiotemporal complexity, which further impedes outbreak prediction efforts [13,14]. The activity of RRV within local reservoir host populations, a potentially significant factor in predicting outbreaks [7], is also incredibly difficult to quantify. In the absence of reliable predictive models for RRV activity, health authorities rely on surveillance programs to provide an early warning of mosquito and pathogen activity [15]. However, these programs can be logistically and financially demanding, and incorporating surveillance data into public health prevention strategies may become even more fraught in the future, as urban development and climate change alter RRV disease dynamics.

## Broader Applications and Future Directions

While the Australian experience with RRV provides valuable insight for the management of other mosquito-borne enzootic pathogens, particularly those associated with urban wildlife, it may be of only limited usefulness for other arthritic alphaviruses, such as CHIKV, in which humans are the predominate reservoir host. The dramatic rise in activity of CHIKV internationally is due primarily to the mosquito–human–mosquito transmission cycle, with anthropophilic mosquitoes, such as *Aedes aegypti* and *Aedes albopictus*, playing critical roles in outbreaks [16]. However, in the case of RRV, it is primarily the enzootic vectors that drive outbreaks of disease, given the primarily wildlife–mosquito–human transmission cycles. Consequently, the RRV system illustrates the importance of pathogen- and region-specific prediction and control measures, with the ecological and environmental variability of RRV resulting in multiple epidemiologies in Australia and limiting the utility of generic prediction and control approaches at the local level [13]. In response to any mosquito-borne disease outbreak risk, local authorities must consider the local aspects of the transmission cycle and the RRV system illustrates the importance of region-specific prediction and control measures.

Future work is required to assess the impact of climate change, increasing human populations, and urban development on RRV disease prevalence. Climate change is expected to increase RRV prevalence by changing weather patterns—including rainfall—expanding mosquito ranges, and lengthening warm periods, which may increase active mosquito periods through an extension of the “mosquito season” [14,17]. The growing Australian population and related urbanization may increase the contact rate between humans and reservoir hosts and vectors, increasing disease risk [18]. This is particularly true where constructed and rehabilitated wetlands, which provide suitable habitats for both mosquitoes and wildlife, are located close to growing residential populations. Significant gaps exist in the understanding of wildlife dynamics; further elucidation of the interactions among the virus, mosquitoes, and wildlife is essential for the development of more effective outbreak prediction models. These trends present a daunting picture for future disease mitigation efforts, but analytical innovations, such as those in geospatial information systems (GIS) [19] and emerging vector and pathogen surveillance systems [15], may assist outbreak prevention and prediction efforts in adjusting to these ecological shifts.

## Conclusion

The unique ecology of RRV as a vector and reservoir host generalist makes it a moving target for health authorities. The variability in disease presentation and disease prevalence between vector and host species, within vector and host species, and in space and time makes the virus extremely ecologically complex and limits the scope of predictive models, which in turn impedes control efforts. The challenges presented by RRV offer a unique example of the importance of understanding the ecological underpinnings of disease systems for effective management.
